# Perioperative Trends in Distress Among Cancer Patients: A Systematic Review and Meta‐Analysis

**DOI:** 10.1002/cam4.70456

**Published:** 2025-03-13

**Authors:** Dana G. Rowe, Ellen O'Callaghan, Seeley Yoo, Juliet C. Dalton, Joshua Woo, Edwin Owolo, Tara Dalton, Margaret O. Johnson, Andrea N. Goodwin, Kerri‐Anne Crowell, Samantha Kaplan, Melissa M. Erickson, C. Rory Goodwin

**Affiliations:** ^1^ Duke University School of Medicine Durham North Carolina USA; ^2^ Department of Neurosurgery Duke University Medical Center Durham North Carolina USA; ^3^ Department of Sociology Carolina Population Center, University of North Carolina at Chapel Hill Chapel Hill North Carolina USA; ^4^ Biostatistics Shared Resource Duke Cancer Institute Durham North Carolina USA; ^5^ Medical Center Library and Archives Duke University Durham North Carolina USA; ^6^ Department of Orthopedic Surgery Duke University Medical Center Durham North Carolina USA

**Keywords:** cancer, distress, HRQOL, oncology surgery, perioperative, quality of life

## Abstract

**Introduction:**

Distress is common among cancer patients, especially those undergoing surgery. However, no study has systematically analyzed distress trends in this population. The purpose of this study was to systematically review perioperative rates of distress, as well as differences across cancer types, in cancer patients undergoing surgical intervention.

**Methods:**

A systematic review was conducted using PubMed, EMBASE, Scopus, and APA PsycINFO (searched until July 17, 2023). Included studies were clinical studies of cancer patients undergoing surgery reporting distress measured by the National Comprehensive Cancer Network (NCCN) distress thermometer (DT). Data on study and patient characteristics, and preoperative and postoperative distress rates were extracted. Results were pooled, and overall distress rates were calculated as weighted means. Subanalysis by cancer type was performed. Three meta‐analyses were conducted: (1) preoperative distress, (2) postoperative distress, and (3) change in distress.

**Results:**

Fifty‐seven studies including 13,410 cancer patients were reviewed. Most patients were female (67.4%), White (77.8%), and married/partnered (72.2%), with an average age of 59.2 years. The most common cancers were breast (14 studies), brain (8), and colorectal (7). Weighted mean pre‐ and postoperative distress scores were 5.1 and 4.5, respectively. Distress remained high through 30 days postoperatively, then declined thereafter. Brain cancer patients reported the highest postoperative distress (5.1), followed by breast cancer patients (4.9).

**Conclusion:**

The perioperative phase is a critical period of elevated distress in cancer patients. Preoperatively, patients experience moderate to severe levels of distress, which persist throughout the early postoperative phase, gradually declining from the 1‐month postoperative mark onwards.

## Graphical abstract

Distress is common among cancer patients. A systematic review of 57 studies found that distress levels are moderate to severe preoperatively and persist through the early postoperative phase, gradually declining from the 1‐month postoperative mark onwards. Notably, patients with brain cancer reported the highest postoperative distress levels, followed by those with breast cancer.

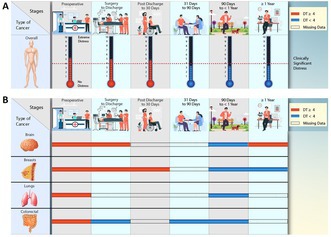

## 1 Introduction

The experience of distress is pervasive among cancer patients, stemming from challenges associated with their diagnosis, treatments, and the broader psychosocial burden of their disease. Studies highlight the prevalence of distress, with up to 61% of cancer patients experiencing clinically significant levels [1, 2, 3]. Surgical intervention, an integral component of cancer care for many patients, further compounds this distress [4, 5]. Notably, elevated distress levels have been consistently associated with worse quality of life and poor patient outcomes, including missed appointments, longer hospital stays, and increased mortality rates [6, 7, 8, 9, 10].

Recognizing the burden of distress in patients with cancer and its profound impact on their well‐being and outcomes, the National Comprehensive Cancer Network (NCCN) created the distress thermometer (DT) as a screening tool to identify psychosocial needs. This self‐reported tool uses a 0–10 scale, identifying scores of 4 or higher as clinically significant levels of distress [10, 11, 12, 13]. Patients are further prompted to identify specific sources of distress using the Problem List component of the DT. Widely adopted and validated across multiple disease types and languages, the tool serves as an essential resource for comprehensive distress evaluation [14, 15, 16, 17].

Prior studies utilizing the DT have demonstrated high rates of distress in surgical cancer patients, evident both before and after surgery [18, 19, 20, 21]. However, variations in the time points at which distress is measured (e.g., at admission, discharge, or multiple years postoperatively), coupled with the dearth of comparisons between preoperative and postoperative distress levels, hinder a comprehensive understanding of the impact of surgery on patients' psychosocial well‐being. Moreover, very few studies have tracked distress trends over time postsurgery, making it difficult to ascertain critical periods of heightened patient distress.

Given the clinical implications of untreated distress, it is imperative to characterize the specific time points during cancer treatment when patients are most vulnerable. Therefore, a systematic review is merited to help delineate perioperative distress trends. This study aimed to assess the preoperative and postoperative distress rates within the surgical oncology patient population. A secondary aim is to explore postoperative distress trends and discern variations in trends across various cancer types. We hypothesized that patient distress will be lower in the postoperative period compared to the preoperative period, with variations in this change based on cancer type.

## 2 Methods

### 2.1 Study Design

A systematic review of the literature was reported using PRISMA (Preferred Reporting Items for Systematic Reviews and Meta‐Analyses) guidelines [22] to assess the following two research questions: (1) Among adults (≥ 18 years) with a diagnosis of cancer undergoing surgical intervention, what are the preoperative and postoperative distress rates? (2) Among adults (≥ 18 years) with a diagnosis of cancer undergoing surgical intervention, are there differences in postoperative distress rates across various cancer types?

### 2.2 Information Sources and Search Strategy

A medical librarian with expertise in systematic searching composed a sensitive search utilizing a mix of keywords and subject headings to represent the concepts of distress thermometers, surgery, and cancer. The databases PubMed, EMBASE, Scopus, and APA PsycINFO were searched from inception until July 17, 2023. Nonhuman studies were removed when possible. All search results were compiled in EndNote 21 and imported into Covidence for deduplication and screening. Reproducible search strategies are available in the Supporting Information (Methods S1). The study was registered in the PROSPERIO International Prospective Register of Systematic Reviews (CRD42023464973).

### 2.3 Eligibility Criteria

The inclusion criteria were the following: (1) clinical study involving patients diagnosed with solid‐organ cancer undergoing surgical intervention, (2) report patient distress based on the NCCN's distress thermometer (3) published in a peer‐reviewed journal, and (4) published in English. The exclusion criteria were the following: (1) patients with “liquid tumors” (leukemia or lymphoma) only, (2) studies with insufficient data, (3) review studies, and (4) abstract only.

### 2.4 Study Selection and Screening

A two‐stage screening process was used to select studies. The literature search was performed by two authors (E.C. and S.Y.), using the inclusion and exclusion criteria outlined above. The titles and abstracts identified in the search were screened, and potentially eligible studies received a full‐text review. In cases of disagreement, consensus was reached through open discussion, detailed review of the full text, and arbitrations by a third author (D.R.).

### 2.5 Data Extraction

The relevant information regarding the study characteristics, including the study design, level of evidence (LOE), methodological quality of evidence (MQOE), patient population, and distress thermometer data, were collected by three authors (D.R., S.Y., and E.O.) using a predetermined data sheet. Specific DT extracted included (1) preoperative and postoperative distress scores, (2) timing of DT screening, (3) location of DT screening, (4) treatment prior to DT screening, (5) DT cutoff point, (6) number of patients meeting designated DT cutoff point, and (7) number of patients with each item selected on the DT Problem List. We did not include multiple studies using the same cohort; instead, only a single study with the largest sample and most complete dataset was included. When studies were assessing the impact of an intervention on distress levels and included both a control and intervention group, distress data exclusively from the control group were utilized to maintain consistency across studies.

### 2.6 Quality Assessment

The authors appraised the quality of the included studies using the Oxford Centre for Evidence‐Based Medicine Levels of Evidence tool [23]. Risk of bias was assessed via the Joanna Briggs Institute Critical Appraisal checklist for studies reporting prevalence data, which uses a 9‐item checklist [24]. An item was scored “0” if it was answered “no” or “unclear” and “1” if it was answered “yes.” Article quality assessment was defined as follows: high‐risk quality = 0–3, moderate risk quality = 4–6, and low risk = 7–9. Quality assessment was performed by two reviewers (D.R. and E.O). In cases of disagreement, consensus was reached through discussion and a detailed review of the full text.

### 2.7 Statistics

Descriptive characteristics of included studies are reported as mean for continuous variables and percent (%) for categorical variables. Where appropriate, results of the included studies were pooled, and the overall rates were subsequently calculated as weighted means with 95% confidence intervals using a random effects model. Three meta‐analyses were conducted and displayed as forest plots (1) preoperative distress, (2) postoperative distress, and (3) change in distress from the preoperative to postoperative period. Descriptive statistics for included studies were computed in Excel using the average, COUNTIF and SUMIF functions. Meta‐analyses were performed in R Studio (version 4.2.2).

## 3 Results

### 3.1 Literature Search and Data Extraction

**FIGURE 1:**
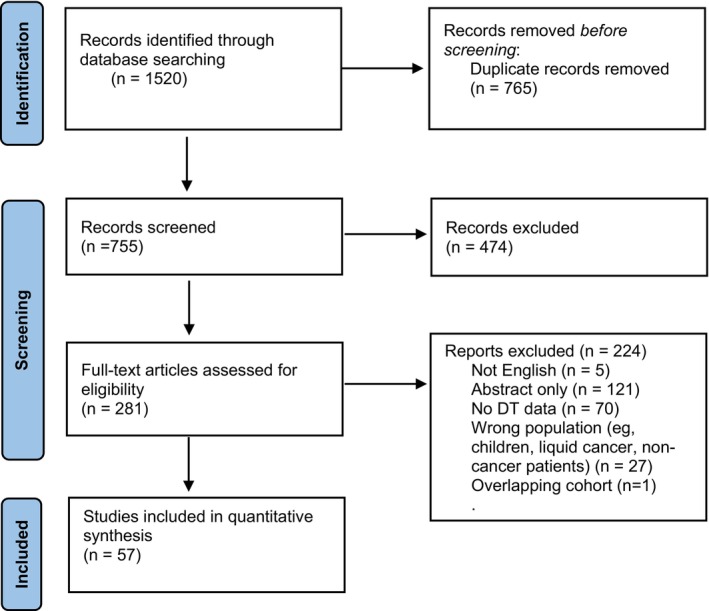
(a) Forest plot of preoperative distress. (b) Forest plot of postoperative distress. (c) Forest plot of change in distress from preoperative to postoperative period.

The initial literature search yielded 755 articles after duplicates were removed. The titles and abstracts were screened, and 474 articles were excluded. The full text of the remaining 281 studies were assessed for eligibility; 224 were excluded. Ultimately, data were extracted from 57 full‐text articles [4, 5, 19, 21, 25, 26, 27, 28, 29, 30, 31, 32, 33, 34, 35, 36, 37, 38, 39, 40, 41, 42, 43, 44, 45, 46, 47, 48, 49, 50, 51, 52, 53, 54, 55, 56, 57, 58, 59, 60, 61, 62, 63, 64, 65, 66, 67, 68, 69, 70, 71, 72, 73, 74, 75, 76, 77]. The PRISMA flow diagram summarizes the selection process (Figure 1).

### 3.2 Study Characteristics and Patient Demographics

The 57 included studies had a combined total 15,566 patients (Table 1). Of these, 13,410 patients reported DT data (Table 1). The weighted mean age of the DT cohort was 59.2 years (range 18–93). The majority of these patients were female (67.4%), married/partnered (72.2%), and white (77.8%). About one third (31.0%) of patients had some higher education. The most common primary cancer across the included studies was breast (14 studies) followed by brain (8 studies) and colorectal cancer (7 studies). Data included in this review were mainly from European (35 [61.4%]), North American (9 [15.8%]), and Asian (6 [10.5%]) cohorts. Prospective cohort studies represent 82.5% (*n* = 47) of the studies, whereas retrospective cohort studies account for 17.5% (*n* = 10). A summary of study characteristics and patient demographics are shown in Table 1, full details can be found in Appendix S1.

**TABLE 1:** Summary of characteristics of included studies.

Study demographics	All studies = 57	
Cohort characteristics	Total cohort	Cohort with DT data reported
Participant characteristics		
Number of patients	15,566	13,410
Sex %^a^		
Male	34.2%	32.6%
Female	65.8%	67.4%
Mean number of patients per study	273.1	235.3
Age at surgery (years)		
Weighted Mean	60.7	59.2
% Married^b^	—	72.2%
% White^c^	—	77.8%
% Higher education^d^	—	31.0%
Study characteristics		
Cohort geography		
Asian	6	—
Australian	5	—
European	35	—
North American	9	—
South American	2	—
Study types		
Prospective cohort	47	—
Retrospective cohort	10	—
Type of cancer		
Bladder	1	—
Bone/soft tissue	2	—
Brain	8	—
Breast	14	—
Colorectal	7	—
Gynecologic	4	—
Gastric	1	—
Head and neck	2	—
Lung	3	—
Skin	1	—
Pancreatic	1	—
Pelvic	2	—
Penile	1	—
Peritoneal	2	—
Prostate	2	—
Renal	1	—
Testicular	1	—
Multiple types of cancer	4	—

^a^ *n* = 13,257 for overall cohort, 9723 for DT cohort.

^b^ *n* = 6602.

^c^ *n* = 2558.

^d^ *n* = 3889.

### 3.3 Distress Thermometer Characteristics

Mean distress thermometer data are reported in Table 2. Preoperative mean DT scores were reported in 25 (42.1%) studies, including 5455 patients. The mean preoperative distress score was 5.1 (SD 2.64). Fifteen studies reported on location of preoperative DT measurement: the majority of DT scores were collected at hospital admission (6 [42.9%] of 14 studies), followed by outpatient preoperative appointments (4 [28.6%] of 14 studies) and at initial diagnosis (4 [28.6%] of 14 studies). Within subanalysis of the most‐reported cancer types, brain tumor patients had the highest weighted mean preoperative distress level at 6.3, followed by colorectal cancer patients (4.8).

**TABLE 2:** Summary of preoperative and postoperative distress thermometer scores.

	Mean distress scores			Distress thermometer cutoff scores			
Cohort	Studies	Patients	Weighted mean	Studies	Cutoff point	Patients	% at or above cutoff
Preoperative							
Overall	25	5455	5.1	20	Mixed	3982	32.6%
Breast	7	2341	5.5	5	4,5,7	2164	59.2%
Brain	2	188	6.3	3	6	206	53.9%
Colorectal	3	1697	4.8	2	4	1822	64.0%
Lung	3	114	4.0	NR	NR	NR	NR
Gynecologic	NR	NR	NR	2	4	712	42.6%
Postoperative							
Overall	27	4903	4.5	21	Mixed	4090	18.0%
Breast	7	1967	4.9	8	4,5,7	1868	34.3%
Brain	7	955	5.1	4	6	774	43.3%
Colorectal	2	327	3.8	1	4	255	59.2%
Lung	2	74	3.4	NR	NR	NR	NR
Gynecologic	NR	NR	NR	1	4	193	51.3%

Abbreviations: NR; not reported.

Postoperative DT scores were reported in 27 (47.3%) studies, including 4903 patients. The average postoperative distress score was 4.5 (SD 2.77). Fifteen studies reported on location of postoperative DT measurement. Most postoperative DT scores were collected in the hospital prior to discharge (9 [60.0%] of 15 studies) followed by outpatient postoperative appointments (6 [40.0%] of 15 studies). Nine studies reported DT scores at multiple postoperative timepoints. The timing of postoperative distress measurement was highly variable, ranging from 24 h to over 5 years after surgery. Brain tumor patients had the highest weighted mean postoperative distress level at 5.1, followed by breast cancer patients (4.9).

### 3.4 Distress Thermometer Cutoffs

Distress thermometer cutoff data are reported in Table 2. Twenty studies (3982 patients) reported on the number of patients meeting a predefined cutoff point to identify moderate‐to‐severe levels of distress. These point cutoffs varied by study, ranging from 4 to 7. Overall, 32.6% of patients met this cutoff preoperatively. Within cancer‐specific subanalysis, we saw higher proportions of patients meeting these cutoffs: 64.0% of colorectal cancer patients met the cutoff, followed by breast (59.2%) and brain (53.9%) cancer.

Twenty‐one studies (4090 patients) reported on the number of patients meeting a predefined cutoff postoperatively, with 18.0% of patients meeting the cutoff overall. Colorectal patients again had the highest proportion of patients meeting the cutoff (59.2%), followed by gynecologic cancer patients (51.3%).

### 3.5 Distress Thermometer Trends Over Time

**FIGURE 2:**
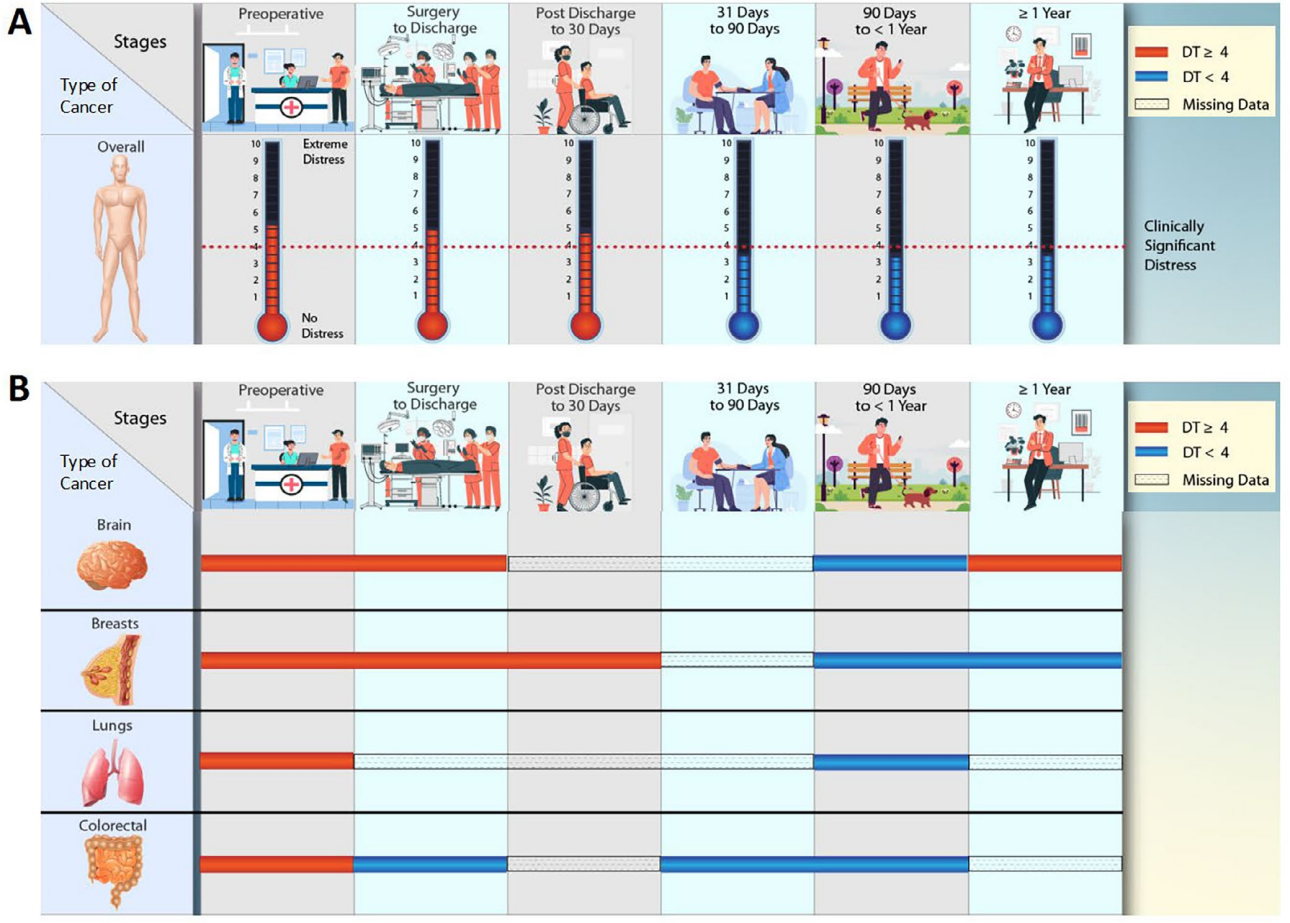
Distress scores by perioperative time point.

Overall, weighted mean distress scores decreased slightly from the preoperative period to the immediate postoperative period (5.1 to 4.9, respectively). Scores stayed elevated above the NCCN's cutoff value of 4, signifying clinically significant distress levels [1], up through the 30‐day postoperative point, at which point the scores began trending down (Figure 2). These patterns varied slightly within specific cancer cohorts, as seen in Table S1.

### 3.6 Meta‐Analysis of Mean Distress Scores

**FIGURE 3:**
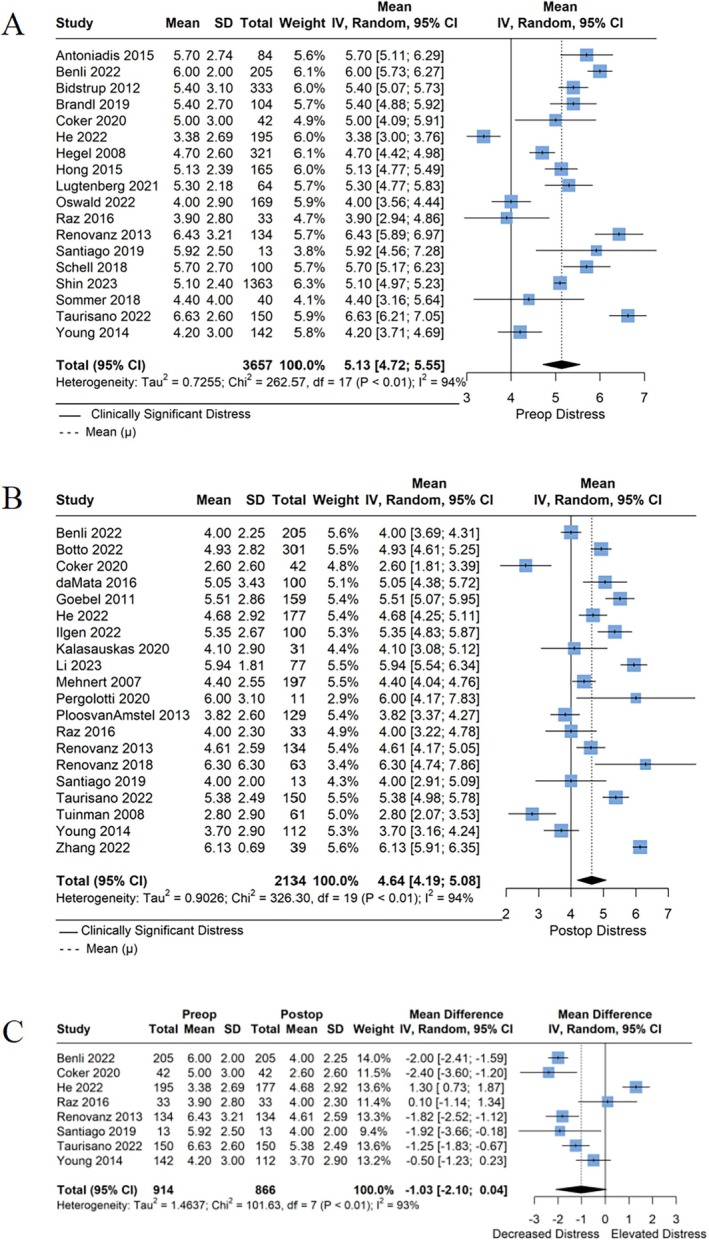
Distress scores by perioperative time point.

Of the 25 studies reporting mean preoperative distress values, 18 reported measures of variance. On meta‐analysis, the mean preoperative distress score was 5.18 (95% confidence interval [CI]: 4.72; 5.55). The forest plot of preoperative distress is presented in Figure 3.

Of the 27 studies reporting mean postoperative distress values, 20 reported measures of variance. On meta‐analysis, the mean postoperative distress score was 4.64 (95% CI: 4.19; 5.08). The forest plot of postoperative distress is presented in Figure 3b.

The meta‐analysis of change in distress included eight studies. The mean change in distress was −1.03 (95% CI: −2.10; 0.04), demonstrating lower postoperative distress scores relative to preoperative scores. The forest plot of change in distress is presented in Figure 3c.

### 3.7 Problem List

**FIGURE 4:**
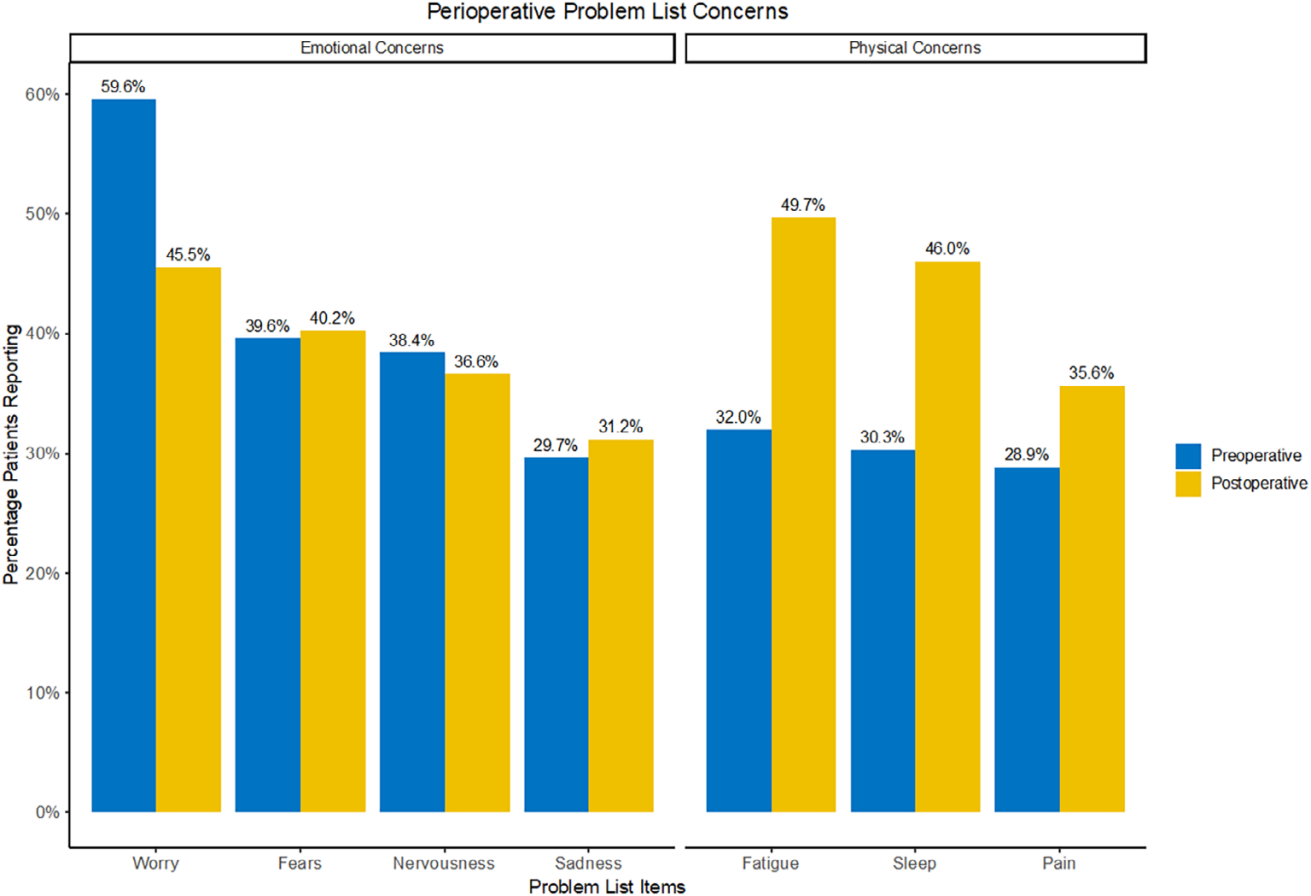
Perioperative problem list concerns.

Ten studies reported preoperative Problem List concerns, and nine studies reported postoperative concerns. Emotional and physical concerns predominated, with fewer patients reporting practical, social, or spiritual concerns. The most common preoperative concern was worry, with 59.5% of patients reporting this concern. Fears (39.6%), nervousness (38.4%), fatigue (32.0%), and sleep (30.3%) were also commonly cited concerns (Table S2 and Figure 4). Postoperatively, the most common concern was fatigue (49.7%), followed by sleep (46.0%), worry (45.5%), and fears (40.2%) (Table S2 and Figure 4). Looking at the change in percentage of patients citing each concern, we observed that emotional concerns tended to decrease postoperatively, whereas physical concerns tended to increase (Table S2 and Figure 4).

### 3.8 Evidence Appraisal

The majority (47 [82.5%]) of the 57 included studies constitute level 2 evidence according to the Oxford Centre for Evidence‐Based Medicine Levels of Evidence. More specifically, most studies were classified as level 2b studies, representing either retrospective symptom prevalence studies or prospective symptom prevalence studies with poor follow‐up. Regarding risk of bias, the majority of studies (43 [75.4%] of 57) were classified as low risk (score 7–9) per the Joanna Briggs Institute Critical Appraisal checklist for studies reporting prevalence data (Appendix S1).

## 4 Discussion

To our knowledge, this systematic review and meta‐analysis represents the first study examining perioperative distress among cancer patients. Our findings highlight surgical intervention and the perioperative period as critical timepoints characterized by heightened distress. Patients exhibit moderate to severe levels of distress preoperatively and remain in this state of heightened distress up to 1 month postoperatively. Only then do we start to see these high levels of distress decline. This insight offers a deeper understanding of the fluctuating nature of distress throughout the perioperative phase for cancer patients.

Elevated distress levels in cancer patients have been linked to diminished quality of life and failure to acknowledge or detect this distress may lead to worse clinical outcomes. Acknowledging the association between distress levels and poor outcomes, the NCCN recommends referring patients who report moderate‐to‐severe distress (scoring 4 or higher on the DT) to appropriate supportive services; however, while the NCCN recommends initial screening at diagnosis and at relevant intervals thereafter, it does not explicitly include surgical intervention in these intervals [78]. Our study findings highlight surgical intervention and the perioperative period as a critical phase of heightened distress, a trend that persisted across various cancer types. These findings offer insights to clinicians and institutions as they implement distress screening policies, underlining the necessity of perioperative screening and targeted interventions.

Our results underscore persistently elevated distress rates within the initial 30 days following surgery. Though surgery may provide patients with some degree of symptom relief, reflected by a mild decrease in overall distress scores, it is important to note that distress is multifactorial. The elevated levels of distress postsurgery suggests that there are other factors beyond the procedure itself contributing to patients' heightened distress postoperatively. The sources of concern noted on the Problem List may help elucidate the factors contributing to postoperative distress.

Prior studies have demonstrated a strong association between pain and psychological distress, and in the immediate postoperative period, pain is likely a contributing factor to heightened distress [79]. This is supported by the observed increase in the number of patients reporting pain on their Problem List after surgery (Figure 3). Furthermore, studies have demonstrated a strong relationship between cancer, insomnia, and fatigue, with the latter being reported in as much as 80% of patients in some cancer types [80]. While postoperative fatigue may be related to the body's response to surgery, postoperative insomnia can be an indirect result of high distress, creating a positive feedback loop of insomnia‐induced distress. These findings are consistent with our results, which highlight a high rate of postoperative fatigue and sleep disturbances (Figure 3). However, additional studies will be needed to elucidate the complex relationships between the physical and emotional drivers of distress in cancer patients.

Inadequate patient–provider communication during the perioperative period may amplify distress, especially if expectations regarding pain management and recovery are unclear [81, 82]. As patients proceed through the recovery process, they may also be anxiously awaiting pathology results related to diagnosis or resection margins. Others may already be anticipating the challenges of the next phase of treatment, including chemotherapy and/or radiation. These uncertainties introduced in the postoperative period have been identified as major sources of anxiety and distress among cancer patients [83]. Moreover, in the 1‐month postoperative period, patients may experience distress from factors like financial stress, new disabilities, caregiver responsibilities, and prolonged absence from work [84]. Therefore, it is crucial for providers to screen and inquire about individual sources of distress to help facilitate the appropriate support services for each patient.

Variations in distress scores observed across different cancer types emphasize the necessity for tailored postoperative screening protocols and personalized discussions regarding sources of distress. Notably, among the cancer cohorts evaluated, patients with brain tumors exhibited the highest levels of both preoperative and postoperative distress. However, in contrast to the marginal decrease in distress observed in the overall cohort postoperatively, patients with brain tumors demonstrated a significant decline in distress levels, from 6.3 preoperatively to 5.3 postoperatively. This trend may be attributed to the relief of distressing symptoms—such as headaches, nausea, seizures, fatigue, and neurological deficits—commonly experienced by brain cancer patients before surgery. Surgical resection often alleviates these symptoms, thus mitigating patient distress. Conversely, the trajectory of distress levels in breast cancer patients displayed a different pattern. Although these patients experienced an initial decline in distress in the immediate postoperative period, they reverted to nearly preoperative distress levels after discharge. This trend may reflect the anxiety associated with awaiting confirmatory results like negative margins and sentinel lymph node biopsy results in the first few weeks postsurgery, coupled with the anticipation of subsequent treatment phases [85]. Additionally, for patients undergoing mastectomies, distress might intensify in the month following surgery due to a perceived loss of identity, impacting patients' sense of femininity and role as women or mothers.^86^ Considering both cancer‐related and surgery‐induced factors is crucial when identifying patients at heightened risk of distress and establishing critical screening timepoints. Understanding these nuances gives clinicians the opportunity to offer targeted interventions and improve patient outcomes.

Our findings regarding distress levels beyond 1‐year postsurgery highlight the complex nature of distress experienced by cancer patients. Notably, we observed heightened distress scores at the 1‐year mark compared to the 90 days to 1‐year postoperative period. These findings persisted across each cancer type in our subanalyses. As time progresses from the initial perioperative period, patients may grapple with heightened concerns surrounding fear of disease progression, prognosis, mortality, potential functional declines, and decisions surrounding end‐of‐life care. Although our study did not specifically assess contributory factors for distress at each timepoint, the observed escalation in distress beyond 1 year underscores the critical necessity for continuous and consistent distress screening and psychosocial support in cancer care. This proactive screening approach enables healthcare providers to remain attuned to their patients' evolving needs, identify emerging mediators of distress, and provide timely intervention throughout the treatment course.

### 4.1 Limitations

This study has several inherent limitations. As a systematic review and meta‐analysis, it inherits the limitations of each included study. Most studies reported only pooled distress means, limiting patient‐level analysis and correlation with disease severity or function. Additionally, meta‐analyses require measures of central tendency and spread, which were not available for all studies, especially for changes between preoperative and postoperative periods. Nonetheless, comparable trends were observed. Many studies excluded patients with cognitive or functional impairments, which may bias results, as these patients might report higher or more variable distress.

The generalizability of our findings is also constrained by the predominantly White, partnered, and younger study population, which may not accurately represent the broader cancer patient demographic. Furthermore, the variety of surgical procedures included introduces heterogeneity that could affect generalizability. Most studies had a low risk of bias, while those with moderate risk were generally small, which minimized their impact on pooled results. Finally, the reliance on retrospective studies with limited follow‐up underscores the need for more prospective research on distress in cancer patients.

Despite these limitations, this review and meta‐analysis provide a valuable overview of the literature and represent the first systematic examination of changes in distress during specific perioperative windows for cancer patients.

### 4.2 Future Directions

Future research should prioritize underrepresented groups, particularly non‐White, nonpartnered, and older patients, to capture distress trends across diverse backgrounds and to develop support strategies that meet unique patient needs. Additionally, studies should investigate specific sources of distress among oncology patients undergoing surgery, as distress trends vary across cancer types. Cancer‐specific research will be essential for tailoring effective interventions. Prospective studies are also needed to evaluate interventions aimed at reducing perioperative distress, as these strategies hold significant promise for enhancing patient experiences and improving clinical outcomes.

## 5 Conclusion

Our investigation of psychosocial distress in cancer patients during the perioperative period and throughout their clinical course of disease emphasizes the complex nature of this challenge. Our findings identify the perioperative period as a critical timepoint of heightened distress levels among cancer patients. Preoperatively, patients experience moderate‐to‐severe levels of distress, which persist throughout the early postoperative phase and gradually decline from the 1‐month postoperative mark onwards. Furthermore, beyond the first year after surgery patients' distress levels began to rise again in the study population. These findings offer a deeper understanding of the fluctuating nature of distress throughout the perioperative phase for cancer patients. Understanding these patterns of distress emphasizes the importance of adopting proactive, targeted screening approaches to better assess the individual needs of cancer patients. Additionally, these findings provide helpful guidance for institutions that aim to establish more concrete protocols for distress screening. Above all, identifying the critical time points for distress screening enables healthcare providers to intervene before adverse outcomes, such as diminished quality of life, increased morbidity and mortality, and prolonged hospital stays, impact a patient's clinical trajectory.

## Author Contributions

**Dana G. Rowe:** conceptualization (equal), data curation (lead), formal analysis (supporting), investigation (equal), methodology (equal), project administration (lead), visualization (equal), writing – original draft (equal), writing – review and editing (equal). **Ellen O'Callaghan:** data curation (equal), formal analysis (equal), investigation (equal), writing – original draft (equal), writing – review and editing (equal). **Seeley Yoo:** data curation (equal), investigation (equal), methodology (equal), validation (equal), writing – original draft (equal). **Juliet C. Dalton:** visualization (equal), writing – original draft (equal), writing – review and editing (equal). **Joshua Woo:** writing – review and editing (equal). **Edwin Owolo:** investigation (equal), validation (equal), writing – original draft (equal). **Tara Dalton:** conceptualization (equal), investigation (equal), writing – original draft (equal). **Margaret O. Johnson:** conceptualization (equal), methodology (equal), visualization (equal), writing – original draft (equal). **Andrea N. Goodwin:** investigation (equal), supervision (equal), writing – original draft (equal), writing – review and editing (equal). **Kerri‐Anne Crowell:** formal analysis (equal), methodology (equal), supervision (equal), validation (equal), writing – original draft (equal). **Samantha Kaplan:** conceptualization (equal), data curation (equal), methodology (equal), writing – original draft (equal), writing – review and editing (equal). **Melissa M. Erickson:** conceptualization (equal), investigation (equal), methodology (equal), supervision (equal), visualization (equal), writing – original draft (equal), writing – review and editing (equal). **C. Rory Goodwin:** conceptualization (equal), data curation (equal), formal analysis (equal), methodology (equal), supervision (equal), validation (equal), writing – original draft (equal), writing – review and editing (equal).

## Disclosure

Dana Rowe: Received grants from Duke Bass Connections, a Pfizer Foundation grant and the Duke Clinical Translational Science Institute (CTSI). C. Rory Goodwin: Received grants from the Robert Wood Johnson Harold Amos Medical Faculty Development Program, the Federal Food and Drug Administration, Duke Bass Connections, and the NIH 1R01DE031053‐01A1. Consultant for Stryker and Medtronic. Deputy Editor for Spine. Patent Application/invention disclosures outside of the current work. Melissa M. Erickson: Received grants from Duke Bass Connections. Consultant for Medtronic, Restor3D, Depuy Synthes, and Globus. Received fellowship funding from Globus/Nuvasive and Medtronic.

## Conflicts of Interest

The authors declare no conflicts of interest.

## Precis

Distress is common among cancer patients and a systematic review of 57 studies found that distress levels are moderate to severe preoperatively and persist through the early postoperative phase, gradually declining from the 1‐month postoperative mark onwards. Notably, patients with brain cancer reported the highest postoperative distress levels, followed by those with breast cancer.

## Supporting information


Data S1.

## Data Availability

Data will be made available upon individual request and at the discretion of the corresponding author.
