# Supplementary material for: Perioperative Trends in Distress Among Cancer Patients: A Systematic Review and Meta‐Analysis
Source: Cancer Med. 2025 Mar 13;14(6):e70456. doi: 10.1002/cam4.70456 (PMC11904430; doi:10.1002/cam4.70456)
Supplement: Supplementary file 1 — Data S1. [file CAM4-14-e70456-s001.docx]

**SUPPLEMENTARY MATERIAL**

| **Appendix 1:** Summary of Included Studies | | |  |  |  |  |  |
| --- | --- | --- | --- | --- | --- | --- | --- |
| **Authors** | **Year** | **No. Patients (DT data)** | **LOE** | **MQOE** | **Female** | **Age (Years)*** | **Cancer Surgery** |
| Admiraal et al. | 2013 | 1010 | 2b | 9 | NR | NR | Multiple |
| Antoniadis et al. | 2015 | 84 | 2b | 8 | 27 | 71 (9.5) | Colorectal |
| Arnaboldi et al. | 2017 | 185 | 2b | 7 | NR | NR | Gynecologic |
| Arnaboldi et al. | 2016 | 154 | 2b | 7 | 128 | 56 (12.3) | Multiple |
| Benli et al. | 2022 | 205 | 2b | 7 | NR | 51 (13.7) | Breast |
| Bidstrup et al. | 2012 | 333 | 2b | 7 | 333 | 60 (10) | Breast |
| Botto et al. | 2022 | 301 | 2b | 8 | 301 | 57 | Breast |
| Brandl et al. | 2019 | 104 | 2b | 8 | NR | NR | Peritoneal |
| Brown et al. | 2020 | 18 | 3b | 4 | 9 | NR | Brain |
| Coker et al. | 2020 | 42 | 1b | 6 | 16 | 61 (13.3) | Pelvic |
| Conduit et al. | 2022 | 39 | 2b | 7 | NR | 32.4 (NR) | Testicular |
| daMata et al. | 2016 | 100 | 2b | 7 | 46 | 60 (13.6) | Multiple |
| Draeger et al. | 2018 | 71 | 2b | 6 | NR | NR | Renal |
| Draeger et al. | 2018 | 280 | 2b | 8 | NR | NR | Bladder |
| Drager et al. | 2017 | 29 | 2b | 7 | NR | NR | Penile |
| Fayanju et al. | 2019 | 1029 | 2b | 8 | 1029 | 58 (48-67)^ | Breast |
| Ghazali et al. | 2017 | 214 | 2b | 6 | NR | NR | Head and Neck |
| Goebel et al. | 2011 | 159 | 2b | 9 | 82 | 55 (15.5) | Brain |
| Hegel et al. | 2008 | 321 | 2b | 8 | 321 | 58 (12.6) | Breast |
| Hoffmann et al. | 2017 | 450 | 2b | 7 | 237 | 51.5 (NR | Brain |
| Hong et al. | 2015 | 165 | 2b | 7 | 38 | 62 (8.9) | Gastric |
| Ilgen et al. | 2022 | 100 | 2b | 7 | 53 | 52 (14.0) | Brain |
| Ise et al. | 2021 | 298 | 2b | 9 | 126 | 53 (18-87) | Bone / Soft Tissue |
| Jansen et al. | 2021 | 34 | 1b | 6 | 24 | 56 (29-78) | Skin |
| Jorgensen et al. | 2016 | 1079 | 2b | 8 | 1079 | 60 (10.8) | Breast |
| Kalasauskas et al. | 2020 | 31 | 2b | 6 | 25 | 57 (13.0) | Brain |
| Kim et al. | 2016 | 41 | 2b | 6 | 23 | 67 (13.0) | Lung |
| Li et al. | 2023 | 77 | 3b | 7 | 38 | NR | Colorectal |
| Lim et al. | 2022 | 31 | 3b | 5 | NR | NR | Colorectal |
| Lugtenberg et al. | 2021 | 64 | 2b | 8 | 64 | 51 (27-71) | Breast |
| Luutonen et al. | 2011 | 268 | 2b | 8 | NR | NR | Breast |
| Mehnert et al. | 2007 | 197 | 2b | 7 | 0 | 66 (5.9) | Prostate |
| Mejdahl et al. | 2015 | 286 | 1b | 7 | 286 | 61 (54-66)^ | Breast |
| Mertz et al. | 2017 | 444 | 2b | 7 | 444 | 61 (51-67)^ | Breast |
| Ohlen et al. | 2019 | 250 | 1b | 8 | 108 | 67 (11.6) | Colorectal |
| Oswald et al. | 2022 | 169 | 2b | 8 | 87 | 55 (13.5) | Peritoneal |
| Pergolotti et al. | 2020 | 11 | 3b | 5 | 11 | 63 (45-74) | Gynecologic |
| Ploos van Amstel et al. | 2013 | 129 | 2b | 6 | NR | 57 (10.0) | Breast |
| Punnen et al. | 2013 | 557 | 2b | 6 | 0 | 60 (6.7) | Prostate |
| Raz et al. | 2016 | 33 | 2b | 6 | 19 | 65.6 (34-91) | Lung |
| Reid et al. | 2022 | 527 | 2b | 8 | NR | 65 (58-70)^ | Gynecologic |
| Renovanz et al. | 2013 | 134 | 2b | 7 | 87 | 53 (NR) | Brain |
| Renovanz et al. | 2018 | 63 | 2b | 7 | 44 | 56 (16.0) | Brain |
| Santiago et al. | 2019 | 13 | 3b | 6 | 8 | 45.8 (12.0) | Bone / Soft Tissue |
| Sarenmalm et al. | 2018 | 459 | 2b | 7 | NR | NR | Colorectal |
| Schaeffeler et al. | 2015 | 189 | 2b | 9 | 206 | 53 (NR) | Breast |
| Schell et al. | 2018 | 100 | 2b | 9 | 43 | 64 (14.7) | Head and Neck |
| Shin et al. | 2023 | 1363 | 2b | 8 | 608 | 61 (11.3) | Colorectal |
| Sommer et al. | 2018 | 40 | 2b | 8 | 24 | 68 (36-85) | Lung |
| Song et al. | 2021 | 255 | 2b | 8 | 92 | 58 (11.6) | Colorectal |
| Staub-Bartelt et al. | 2022 | 54 | 2b | 6 | 28 | 56 (NR) | Brain |
| Taurisano et al. | 2022 | 150 | 2b | 9 | 150 | 59 (13.2) | Breast |
| Tuinman et al. | 2008 | 61 | 2b | 7 | NR | NR | Multiple |
| Yavuz et al. | 2022 | 193 | 2b | 9 | NR | NR | Gynecologic |
| Yeo et al. | 2023 | 230 | 3b | 7 | NR | NR | Pancreatic |
| Young et al. | 2014 | 148 | 2b | 9 | 55 | 60 (27-81) | Pelvic |
| Zhang et al. | 2022 | 39 | 2b | 8 | NR | 51 (8.0) | Breast |
| *Age reported as Mean (SD) or Median (Range) ^IQR rather than range | | | | | | | |

**eMethods 1: Database Search Strategy**

Database (including vendor/platform): MEDLINE (via PubMed)

| **Set #** | **Search Strategy** | **Results** |
| --- | --- | --- |
| **1. Distress** | **(("Psychological Distress"[Mesh] OR "Stress, Psychological"[Mesh] OR Distress*[tiab]) AND (thermometer[tiab] OR thermometers[tiab]))** | **1093** |
| **2. Surgery** | **"surgery"[sh] OR surg*[tiab] OR operat*[tiab] OR procedur*[tiab]** | **5,054,499** |
| **3. Cancer** | "Neoplasms"[Mesh] OR neoplas*[tiab] OR cancer*[tiab] OR tumor[tiab] OR tumors[tiab] OR tumours[tiab] OR tumour[tiab] OR malignan*[tiab] OR carcinom*[tiab] OR melanom*[tiab] OR sarcom*[tiab] OR leukem*[tiab] OR leukaem*[tiab] OR adenocarcinoma*[tiab] OR chondrosarcom*[tiab] OR osteosarcom*[tiab] OR rhabdomyosarcom*[tiab] OR plasmacytom*[tiab] OR histiocytom*[tiab] OR mesotheliom*[tiab] | **5,153,101** |
| **4. Combination** | **1 AND 2 AND 3** | **210** |
| **Validation String** | 33119788 OR 30167085 OR 31839734 OR 34980291 OR 36728535 OR 36648262 OR 31120575 | **7/7** |

Database (including vendor/platform): Embase via Elsevier

| **Set #** | **Search Strategy** | **Results** |
| --- | --- | --- |
| **1. Distress** | **'distress thermometer'/exp OR ((Distress*:ti,ab) AND (thermometer:ti,ab OR thermometers:ti,ab))** | **2527** |
| **2. Surgery** | **'surgery'/exp OR surg*:ti,ab OR operat*:ti,ab OR procedur*:ti,ab** | **8910438** |
| **3. Cancer** | 'neoplasm'/exp OR neoplas*:ti,ab OR cancer*:ti,ab OR tumor:ti,ab OR tumors:ti,ab OR tumours:ti,ab OR tumour:ti,ab OR malignan*:ti,ab OR carcinom*:ti,ab OR melanom*:ti,ab OR sarcom*:ti,ab OR leukem*:ti,ab OR leukaem*:ti,ab OR adenocarcinoma*:ti,ab OR chondrosarcom*:ti,ab OR osteosarcom*:ti,ab OR rhabdomyosarcom*:ti,ab OR plasmacytom*:ti,ab OR histiocytom*:ti,ab OR mesotheliom*:ti,ab | **7349199** |
| **4. Combination** | **1 AND 2 AND 3** | **588** |
|  | **#1 AND #2 AND #3 AND [humans]/lim** | **552** |

Database (including vendor/platform): Scopus via Elsevier

| **Set #** | **Search Strategy** | **Results** |
| --- | --- | --- |
| **1. Distress** | **TITLE-ABS-KEY( Distress* AND (thermometer OR thermometers))** | **1208** |
| **2. Surgery** | **TITLE-ABS-KEY( surg* OR operat* OR procedur*)** | **12792372** |
| **3. Cancer** | TITLE-ABS-KEY( neoplas* OR cancer* OR tumor OR tumors OR tumours OR tumour OR malignan* OR carcinom* OR melanom* OR sarcom* OR leukem* OR leukaem* OR adenocarcinoma* OR chondrosarcom* OR osteosarcom* OR rhabdomyosarcom* OR plasmacytom* OR histiocytom* OR mesotheliom*) | **6449043** |
| **4. Combination** | **1 AND 2 AND 3** | **341** |

Database (including vendor/platform): APA PsycINFO via EBSCO

| **Set #** | **Search Strategy** | **Results** |
| --- | --- | --- |
| **1. Distress** | **(("DE "Distress" OR (TI Distress* OR AB Distress*)AND ((TI thermometer OR AB thermometer) OR (TI thermometers OR AB thermometers))** | **6594** |
| **2. Surgery** | **(DE "Surgery") OR (TI surg* OR AB surg*) OR (TI operat* OR AB operat*) OR (TI procedur* OR AB procedur*)** | **396927** |
| **3. Cancer** | (TI neoplas* OR AB neoplas*) OR (TI cancer* OR AB cancer*) OR (TI tumor OR AB tumor) OR (TI tumors OR AB tumors) OR (TI tumours OR AB tumours) OR (TI tumour OR AB tumour) OR (TI malignan* OR AB malignan*) OR (TI carcinom* OR AB carcinom*) OR (TI melanom* OR AB melanom*) OR (TI sarcom* OR AB sarcom*) OR (TI leukem* OR AB leukem*) OR (TI leukaem* OR AB leukaem*) OR (TI adenocarcinoma* OR AB adenocarcinoma*) OR (TI chondrosarcom* OR AB chondrosarcom*) OR (TI osteosarcom* OR AB osteosarcom*) OR (TI rhabdomyosarcom* OR AB rhabdomyosarcom*) OR (TI plasmacytom* OR AB plasmacytom*) OR (TI histiocytom* OR AB histiocytom*) OR (TI mesotheliom* OR AB mesotheliom*) | **93477** |
| **4. Combination** | **S1 AND S2 AND S3** | **139** |

| **eTable 1: Mean Distress Scores by Perioperative Time Point** | | | | | |
| --- | --- | --- | --- | --- | --- |
| **Time Point** | ***Overall*** | **Breast** | **Brain** | **Colorectal** | **Lung** |
| Pre-Op | *5.1* | 5.5 | 6.3 | 4.8 | 4.0 |
| Surgery to D/C | *4.9* | 4.9 | 5.3 | 3.1 | NR |
| D/C to 30 days | *4.6* | 5.4 | NR | NR | NR |
| 31 to 90 days | *3.3* | NR | NR | 3.5 | 2.9 |
| 91 days to < 1 year | *3.2* | 1.1 | 3.9 | 2.4 | NR |
| ≥ 1 year | *3.3* | 3.8 | 4.1 | NR | 4.0 |
| Pre-Op; pre-operative, D/C; discharge, NR; not reported | | | | | |

| **eTable 2:** Summary of Problem List Concerns | | |  |  |
| --- | --- | --- | --- | --- |
| **Problem List Items** | **Preoperative** | | **Postoperative** | |
|  | **Denominator** | **% Reporting^a^** | **Denominator** | **% Reporting^a^** |
| ***Emotional Concerns*** |  |  |  |  |
| Worry | 2722 | 59.6% | 496 | 45.5% |
| Fears | 2185 | 39.6% | 406 | 40.2% |
| Nervousness | 1217 | 38.4% | 406 | 36.6% |
| Sadness | 2395 | 29.7% | 406 | 31.2% |
| ***Physical Concerns*** |  |  |  |  |
| Fatigue | 2625 | 32.0% | 548 | 49.7% |
| Sleep | 2450 | 30.3% | 247 | 46.0% |
| Pain | 2722 | 28.9% | 707 | 35.6% |
| ^a^Percentage of patients selecting that specific concern on the Problem List component of their Distress Thermometer (DT) | | | | |
